# Leveraging Hierarchical Population Structure in Discrete Association Studies

**DOI:** 10.1371/journal.pone.0000591

**Published:** 2007-07-04

**Authors:** Jonathan Carlson, Carl Kadie, Simon Mallal, David Heckerman

**Affiliations:** 1 Machine Learning and Applied Statistics Group, Microsoft Research, Redmond, Washington, United States of America; 2 Department of Computer Science and Engineering, University of Washington, Seattle, Washington, United States of America; 3 Center for Clinical Immunology and Biomedical Statistics, Royal Perth Hospital, Perth, Australia; North Carolina State University, United States of America

## Abstract

Population structure can confound the identification of correlations in biological data. Such confounding has been recognized in multiple biological disciplines, resulting in a disparate collection of proposed solutions. We examine several methods that correct for confounding on discrete data with hierarchical population structure and identify two distinct confounding processes, which we call coevolution and conditional influence. We describe these processes in terms of generative models and show that these generative models can be used to correct for the confounding effects. Finally, we apply the models to three applications: identification of escape mutations in HIV-1 in response to specific HLA-mediated immune pressure, prediction of coevolving residues in an HIV-1 peptide, and a search for genotypes that are associated with bacterial resistance traits in *Arabidopsis thaliana*. We show that coevolution is a better description of confounding in some applications and conditional influence is better in others. That is, we show that no single method is best for addressing all forms of confounding. Analysis tools based on these models are available on the internet as both web based applications and downloadable source code at http://atom.research.microsoft.com/bio/phylod.aspx.

## Introduction

There is now clear recognition across several application areas that population structure can confound the statistical identification of associations. An area where this problem was recognized early is the identification of coevolving traits. Felsenstein described the problem and proposed a solution for quantitative traits [1], while Ridley [2] and Maddison [3] described early solutions for discrete traits. A specific application area involving discrete traits is the identification of coevolving amino acids. Numerous researchers recognized the problem and proposed solutions [4]–[11]. Yet another area of application that recognized the problem is the identification of Human Leukocyte Antigen (HLA) alleles that mediate mutation of Human Immunodeficiency Virus type 1 (HIV-1) to escape T-cell epitopes. Recently, Bhattacharya et al. [12] demonstrated the importance of correcting for population structure in this domain. In all these application areas, the population structure arises due to the phylogenetic relationships among the species or individuals being studied.

Another important application area where population structure can confound the identification of associations is genome-wide association (GWA) studies [13], [14]. Although a number of candidate disease genes have been identified in this manner, there has been a lack of reproducibility due in part to confounding by common descent [15], [16]. In these studies, the organisms studied typically reproduce sexually, and hence population structure is not due to phylogenetic relationships. Nonetheless, there is structure due to non-random mating [17], [18], and such structure may be hierarchical [19]–[22] especially in self-mating organisms such as *Arabidopsis thaliana* [23]. Numerous solutions have been proposed including genomic control [24], [25], structured association [26], and other methods [27]–[30]. Several solutions address confounding due to hierarchical structure [23], [31], [32].

In this paper, we examine in detail several methods that correct for confounding on discrete data with hierarchical population structure. To introduce these methods, let us consider how confounding may arise when the population structure is due to phylogenetic relationships. In particular, consider the problem of identifying HLA-mediated immune pressure on HIV-1 intrahost adaptation. Moore *et al.* [33] have suggested that the immune pressure from the cellular arm of the immune system—in particular, CD8+ T cells interacting with MHC-I epitopes presented on the surface of infected cells—can exert substantial selection pressure on HIV. If a particular HLA allele exerts pressure on a certain amino acid in the HIV-1 genome, then we would expect to see a correlation between whether an HIV sequence has that amino acid and whether the patient infected with that sequence has the HLA allele. To determine whether these two variables are associated, we could count the number of patients with and without the amino acid and with and without the allele and apply a simple statistical test such as Fisher's exact test. This procedure, however, ignores the phylogenetic structure among the sequences.

**Figure 1:**
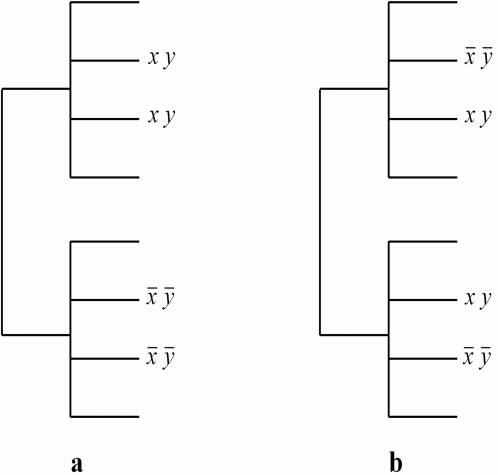
Examples illustrating the (a) overcounting and (b) undercounting of evidence for an association between *X* and *Y*.

Suppose these sequences have the phylogeny shown in Figure 1a. In essence, there are two clusters of sequences where sequences within a cluster are similar to each other but quite different from those in the other cluster. Now suppose we observe that HLA allele *X* and amino acid *Y* are present in two sequences on the upper cluster and absent in the two sequences on the lower cluster, as shown in Figure 1a. The observations of the amino acid are well explained by the phylogeny alone and should not be treated as independent observations. Consequently, the application of Fisher's exact test or some other test that ignores the phylogenetic structure would overcount these observations when determining the correlation of *X* and *Y*. Such overcounting could produce false positives. In contrast, suppose our findings are as shown in Figure 1b. Here, observing the presence and absence of the amino acid in the same branch of the phylogeny is quite surprising, until the observations of *Y* are taken into account. In this case, the application of a simple test would undercount the observations when determining the correlation of *X* and *Y* and potentially produce false negatives—that is, phylogenetic structure helps us to leverage the data to determine more associations than we would have otherwise.

Simple statistical methods such as Fisher's exact test assume the data to be infinitely exchangeable or independent and identically distributed (IID). Although sequence data and other biological data are IID a priori, they are not IID once we learn their hierarchical structure. Furthermore, as we have just seen, this structure can easily confound the statistical search for associations within such data.

An important point that has not been emphasized in previous work is that different applications may involve different evolutionary processes leading to different kinds of confounding and requiring different solutions. For example, in one process, *X* and *Y* coevolve according to the phylogenetic tree—any change in *Y* during evolution influences the evolution of *X*, and vice versa. Here, the phylogenetic tree serves as a confounder of *X* and *Y* in the traditional sense—the tree is a hidden common cause of both *X* and *Y*, leading to spurious correlations between *X* and *Y* when the tree is ignored. In another process, only *Y* evolves according to the tree and the influence of *X* on *Y* occurs only at the tips of the tree. *X* need not evolve according to the tree or “follow the tree,” but instead can have any distribution, including one in which the observations of *X* are IID. We refer to these two processes as *coevolution* and *conditional influence*, respectively.

Both processes lead to confounding, as evidenced by the example in Figure 1, which is agnostic about whether *X* follows the tree. Each process, however, leads to a particular form of confounding that, as we will show, requires a specific solution. In this paper, we use a particular statistical model class known as a *generative* model both to describe these processes and to address each form of confounding that they entail. We describe the *undirected joint model* of Pollock, Taylor and Goldman [5], which represents the traditional confounding process where both *X* and *Y* follow the tree, and our own *conditional model* introduced in Bhattacharya *et al.* [12], which represents the confounding process where only *Y* follows the tree. We show how these models can be used to identify correlations among data when either form of confounding is present. The basic idea behind the approach is to determine the degree to which a generative model that includes a correlation between *X* and *Y* explains the data better than a model that uses only the phylogeny to explain the data.

Using synthetic data, we show that the coevolution model better addresses the confounding of the coevolution process, and the conditional model better addresses the confounding of the conditional process. In addition, we apply these two models to real examples, including the identification of escape mutations in HIV-1 in response to specific HLA-mediated immune pressure and the prediction of coevolving residues in an HIV-1 peptide, and find that no one model is best for all applications.

So far, we have considered only phylogeny as a source of hierarchical population structure. Nonetheless, we also will explore the use of these generative models, which incorporate evolutionary processes, to address hierarchical population structure in its more general case. In particular, we apply these models to a genomic search for genotypes that are associated with bacterial resistance traits in *Arabidopsis* and show that they are effective.

We have implemented methods for fitting these models in a package called PhyloD, which is available on the internet as both a web based application and downloadable source at http://atom.research.microsoft.com/bio/phylod.aspx.

## Results

### Overview of models

The models that we describe are generative models, also known as directed acyclic graphical models [34]. A generative model consists of (1) a *structure*, a directed acyclic graph where nodes correspond to variables and missing arcs specify probabilistic independencies among the variables, and (2) a set of conditional probability distributions, one distribution for each node. The probabilistic independences specified by the structure of the graph allow the joint distribution of the data to be written as the product over the nodes of their conditional distributions. Given the structure of a generative model, we can use observations for some (and not necessarily all) nodes to infer the distributions (or parameters of the distributions) of the model [34]. One common criterion for inference, which we use here, is to identify the distributions that maximize the likelihood of the data.

To represent the hierarchical population structure, our generative models use the same machinery as that found in the maximum-likelihood phylogenetic tree [35]. As we have discussed, however, such hierarchical structure may not arise due to phylogenetic relationships. Also, we note that the trees are built from sequence data alone (see Methods and Discussion).

#### Single-variable model

**Figure 2:**
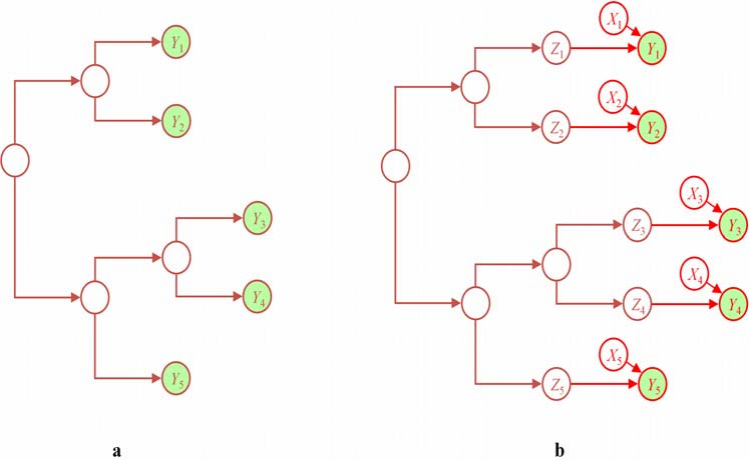
Two generative (graphical) models. (a) The single-variable model for *Y*. (b) The conditional model for *Y* given *X*. The variable *Z_i_* represents the variable *Y_i_* had there been no influence from *X_i_*. Observed variables are shaded. Conditional probability distributions are not shown.

Before we consider models of associations between variables, let us consider the situation where a *single* variable *Y* with possible states present (*y*) and absent (*y̅*) is generated by a given phylogenetic tree. The structure of the generative model, which is simply the series of branches in the phylogenetic tree, is shown in Figure 2a. Nodes at the tips of tree, labeled *Y*_1_, … , *Y_N_* correspond to the observations of *Y*. Unlabeled nodes in the interior of the tree correspond to events of divergence. (Although each *Y_i_* and interior node corresponds to a variable in the ordinary statistical sense, we sometimes refer to the collection of variables—*Y* in this case—as a variable. The proper meaning of the word should be clear from context.) As in the probabilistic model of Felsenstein [35] for a phylogenetic tree, we assume that the transition from state *i* to state *j* along a transition with branch length *t* will occur with probability **P** *_ij_*(*t*) that is determined by a continuous time Markov process (CTMP). In our single binary variable case, the CTMP is parameterized by a single rate parameter λ, and the stationary probability π, the probability of *Y = y* reached by the Markov process after an infinite amount of time. The instantaneous rate of change from *y̅* to *y* is λ π, yielding a transition probability matrix whose values as a function of time *t* are given by
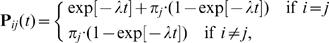
(1)Where π*_j_* = π when *j* corresponds to the presence of *Y*, and π*_j_* = 1−π when *j* corresponds to the absence of *Y*.

As mentioned, given a set of observations for *Y*_1_, … , *Y_N_* at the tips of a tree, we choose the parameters π and λ to be those that maximize the likelihood of the data. Given values for these parameters, the tree structure ψ (the series of branches and their lengths), and the observed data *D_Y_*, the likelihood *L*(*D_Y_*|π, λ, Ψ) can be easily computed [35]. There are a number of methods for identifying the maximum-likelihood parameters, including gradient decent and the Expectation-Maximization (EM) algorithm. For this model and the others that we describe, we use coordinate descent, iteratively maximizing the likelihood for each parameter individually using Brent's method [36].

This generative model is reversible, since π*_i_* **P** *_ij_*(*t*) = π*_j_* **P** *_ji_*(*t*), allowing us to work with an unrooted (or arbitrarily rooted) tree. Also, this model includes the situation where the observations of *Y*_1_, … , *Y_N_* are IID as a special case (i.e., the limit as λ tends to infinity.)

#### Undirected joint model

Now let us consider two binary variables *X* and *Y* that evolve according to a given phylogenetic tree. Our first association model for *X* and *Y*, called the *undirected joint model*, assumes that *X* and *Y* coevolve according to the tree such that, throughout the course of evolution, a change in either variable can influence the evolution of the other. This generative model can be thought of as a single-variable model for a variable *XY* representing the cross product of *X* and *Y* with the four states: *xy*, *xy̅*, *x̅* *y*, 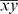. The undirected joint model has five parameters: two rate parameters λ*_X_* and λ*_Y_*, and three parameters π*_xy_*, π*_xy̅_*, π*_x̅_*_*y*_, and 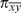 representing the stationary distribution of *XY*. The instantaneous rate matrix *Q* is given by
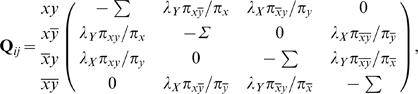
(2)where the diagonal entries assure that the rows sum to 0. Given a particular branch with length *t*, we can compute the probability that one instance of *XY* transitions to another by determining **P** = exp[**Q** *t*], which can be computed using standard numerical Eigen-decomposition techniques [36].

This model, first presented by Pollock and colleagues [5], treats *X* and *Y* symmetrically so that the influence of *X* on *Y* and *Y* on *X* is “undirected,” hence our name for the model. In Methods, we describe an alternative model for coevolution in which the relationship between the two variables is asymmetric or directed.

To determine the significance or strength of the correlation between *X* and *Y*, we determine how much better this model explains the data than a null model in which each variable independently follows the single-variable model described in the previous section. We use both frequentist and Bayesian methods to determine the degree to which the non-null model better explains the data. In the frequentist case, we compute a *p*-value using a likelihood ratio test, which is justified as the null model is nested within the non-null model. We control for multiple tests using *q*-values, which are estimates of one minus positive predictive value (see Methods). In the Bayesian case, we use the Bayesian Information Criterion (BIC) to select the alternative model over the null (see section on model evaluation and comparison).

#### Conditional model

In some situations, it may be reasonable to assume that *Y* follows a tree, but that *X* does not follow the same tree (or any tree at all), and that the influence of *X* on *Y* occurs only at the tips of the tree. In this situation, we sometimes refer to *Y* as the target variable and *X* as the predictor variable. This situation may arise if, for example, *Y* represents the amino acid at a particular position in a sequence and *X* represents an environmental variable that we suspect may be a source of selective pressure on *X*, as in the case where we are looking for HLA alleles that exert pressure on specific HIV-1 molecules to select for escape mutations. Whereas the amino acid likely follows the phylogenetic tree of the protein it is in, it is unreasonable to expect the HLA allele to follow that same tree (except perhaps at the clade level, where HLA alleles may be distributed similarly to the clades of the HIV species tree due to similar geographic constraints). Furthermore, although we expect that HLA pressure acts continually throughout evolution, this pressure could be distributed randomly along the evolutionary paths, and consequently the influence of the known HLA of the patient would only appear at a tip of the tree.

We present a model that captures this notion in Bhattacharya *et al.* [12]. The model is a single-variable model for the target variable *Y*, modified to include the influence of *X* at the tips of the tree. Consider the single-variable model for *Y* as shown in Figure 2a. To construct the conditional model for *Y* given *X*, shown in Figure 2b, we first change each *Y_i_* to *Z_i_*, which represents what *Y_i_* would have been had there been no influence from *X_i_*. We then make each *Y_i_* a probabilistic function of *X_i_* and *Z_i_*, encoding this function with a probability matrix. There are four possible single-parameter transition matrices:

1. **Escape** *X* = *x* increases the probability of *y*→*y̅* transitions,
2. **Attraction** *X* = *x* increases the probability of *y̅*→*y* transitions,
3. **Reversion** *X* = *x̅* increases the probability of *y̅*→*y* transitions,
4. **Repulsion** *X* = *x̅* increases the probability of *y*→*y̅* transitions.

As in the undirected joint case, we can use both frequentist (likelihood ratio test) and Bayesian (BIC) methods to determine the degree to which each of these models better explains the data for *Y* than does the single-variable model for *Y*. Note that we do not need to consider the explanation of the data for *X*, as the model is a conditional one.

Although it is possible that, for example, both escape and reversion processes are acting at the same time, we have found that allowing for two or more processes at the same time dramatically reduces the power of these models. Consequently, in our experimental evaluations, for any given pair of variables, we choose one model from the set above that best explains the data for those variables. We can use both frequentist and Bayesian methods for making this choice. In the frequentist case, we choose the model with the lowest *q*-value. In the Bayesian case, we choose the model with the largest BIC score.

The conditional model is reversible in the sense that the choice of root node among non-tip nodes does not affect the likelihood of the data. We also note that this model is a (discrete) mixed-effects model, wherein the predictor variables *X_i_* correspond to the fixed effects and the hidden variables *Z_i_* correspond to the random effects [37].

### Model evaluation and comparison

We evaluate and compare our models on both synthetic and real data sets. On synthetic data, we examine two questions. One, does taking hierarchical population structure into account improve the analysis? For example, when we generate data from an undirected joint or conditional model, do these models perform better than Fisher's exact test (FET), which assumes the data to be IID? Two, when we generate data from an undirected joint model, does that model better fit the data than the conditional model, and vice-versa?

We use two criteria to measure the performance of a model. First, we measure the ability of the model to discriminate true from false correlations via plots of true positive rate versus one minus positive predictive value. In particular, we compute a *q*-value for every pair *X–Y* that we test, and deem every test with a *q*-value less than some threshold *q*_0_ to be a real association. We then use the known true and false associations in the synthetic data to compute true positive rate and one minus positive predictive value for that threshold. Finally, we allow *q*_0_ to vary, producing a *discrimination curve*. We compute the significance of the difference between two curves by comparing the difference in the areas under the curves against a null model estimated by a permutation test (see Methods). Second, we measure the *calibration* of the model—that is, the degree to which the *q*-value is a good estimate of one minus the positive predictive value. Note that the discrimination curves are independent of calibration, as any monotonic transformation of the threshold used to compute a curve (*q*-values in our case) leaves the curve unchanged.

On real data, we examine whether the undirected joint model or conditional model better represents the data. Because we don't know whether a discovered association is real or not, we cannot use the discrimination curve or calibration criterion to evaluate performance. Furthermore, because neither model is nested within the other, we turn to Bayesian methods—in particular, the Bayesian Information Criterion (BIC)—for comparison. The BIC score for a model with maximum-likelihood parameters θ̂ and *d* free parameters is given by 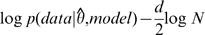. The BIC is an asymptotic approximation to the marginal log likelihood of a model, a Bayesian measure of how well the model represents the data.

To compare the conditional model, a model for *Y* given *X*, with the joint model, a model for *X* and *Y*, we compare the sum of the overall BIC scores for the conditional model and the single-variable model for *X* with the overall BIC score for the joint model. The overall BIC score for the conditional model is the highest BIC score among the conditional models (escape, attraction, reversion, and repulsion) and the independence model. The overall BIC score for the undirected joint model is the higher of the BIC scores for the undirected joint model and the independence model. To test whether there is a significant difference between the overall scores for the conditional and undirected joint models, we apply a two-sided, paired Wilcoxon test to these scores across all *X–Y* pairs in the data.

In our applications, we sometimes find it useful to test whether a variable *Y follows a given tree*. To do so, we calculate the difference in BIC scores for the ordinary single-variable model for *Y* and a model that assumes the observations of *Y*_1_, … , *Y_N_* are IID (a single-variable model for *Y* with λ→∞). We say that *Y* follows the tree if the difference in scores is greater than zero.

### Experiments with synthetic data

We constructed two synthetic data sets, one generated by the process of conditional influence (i.e., by a conditional model) and the other generated by coevolution (i.e., the undirected joint model). The data sets representing conditional influence and coevolution were patterned after the real data sets of Application 1 (effects of immune pressure on HIV mutation) and Application 2 (pairwise amino-acid correlations), respectively. In particular, the sample size (*N*) and number of tests in the data sets were those of the corresponding applications. In addition, the number of variable pairs that were and were not correlated was also patterned after the real data, and the parameters of the generating model for conditional influence (coevolution) were taken from those of a conditional (undirected joint) model fit to the real data. The predictor (HLA) observations for the data set reflecting conditional influence were generated from an IID model.

**Figure 3:**
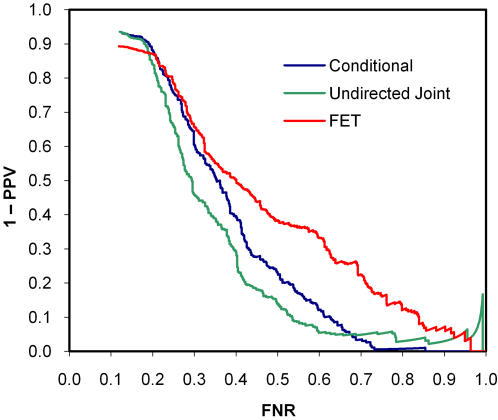
Discrimination curves for synthetic coevolution data. The data closely resemble pairwise amino-acid association data (Application 2).

**Figure 4:**
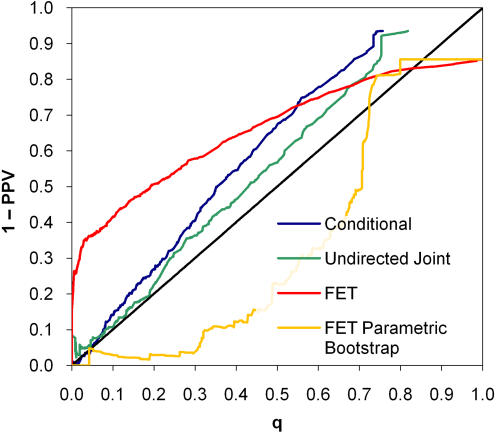
Calibration of *q*-values on synthetic coevolution data. Computing *q*-values for Fisher's exact test using parametric bootstrap results in poor calibration.

**Figure 5:**
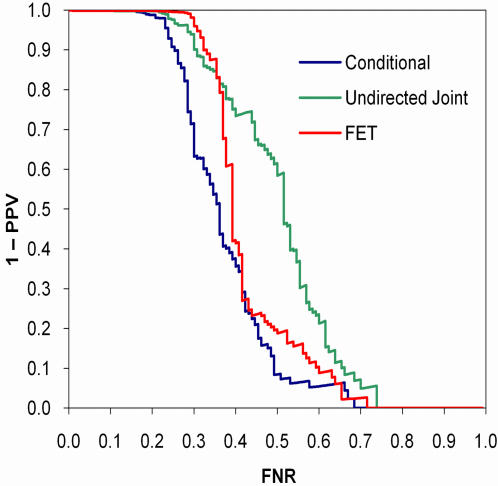
Discrimination curves for synthetic conditional influence data. The data closely resemble the HLA-amino-acid association data (Application 1).

**Figure 6:**
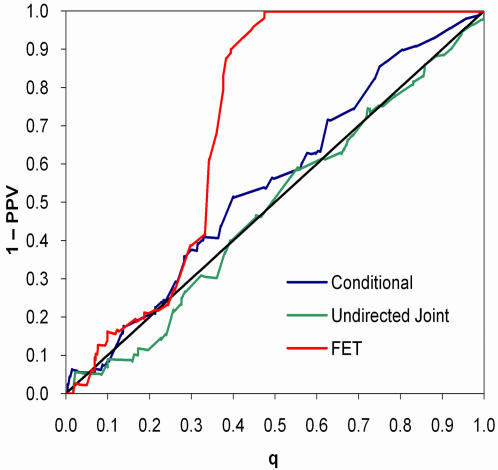
Calibration of *q*-values on synthetic conditional influence data.

As expected, when the data were generated according to the undirected joint model, the joint model was most discriminating, followed by the conditional model (*p* = 0.042) and FET (*p*<0.0001; Figure 3). The *q*-value calibration of the joint model was slightly better than the conditional model, whereas FET was poorly calibrated (Figure 4). Conversely, when the data were generated according to the conditional model, the conditional model was most discriminating, followed by FET (*p* = 0.12) and the undirected joint model (*p* = 0.0013; Figure 5). Both the conditional and joint models were well calibrated, whereas the *q*-values of FET tended to overestimate one minus positive predictive value (Figure 6). Thus, not only did taking hierarchical population structure into account improve the analysis, but the two models could distinguish between the processes of coevolution and conditional influence.

In both examples, the *q*-values from FET were liberal—that is, the *q*-values underestimated the false positives rates. Although we always expect liberal estimates when the data are generated by coevolution because the tree is a hidden common cause of *X* and *Y*, when only one variable follows the tree, the variance of FET's *p*-values will increase due to over- and under-counting, leading to an unpredictable *q*-value calibration. Indeed, on other data sets, we have seen FET produce both liberal and conservative estimates of one minus positive predictive value.

To validate our use of BIC to evaluate model performance on real data, we compared the BIC scores of the undirected joint and conditional models on the synthetic data sets representing conditional influence and coevolution. As expected, we found that the conditional model had a significantly higher score than the undirected joint model on the conditional-influence data set (*p* = 7.6×10^−53^, *N* = 157317), and the joint model performed better model on the synthetic coevolution data set (*p* = 0.06, *N* = 10025).

Finally, we note that, over a wide variety of data sets, the conditional model runs an order of magnitude faster than the undirected joint model, which requires optimization over a larger number of parameters and more complex Eigen decompositions.

#### Sensitivity to tree structure

Our approach raises the question of how sensitive the results are to the structure of the tree used by the models. To address this question, we ran the conditional model on the synthetic conditional influence data using four different trees: the tree used to generate the data (*T_gen_*), a tree with the same structure as *T_gen_* but with the leaf-to-patient assignments randomized (*T_rand_*), and two trees reconstructed from the synthetic amino acids using either a generalized maximum likelihood method (*T_ML_*, the method we use throughout this work ) or a naïve parsimony method (*T_pars_*).

**Figure 7:**
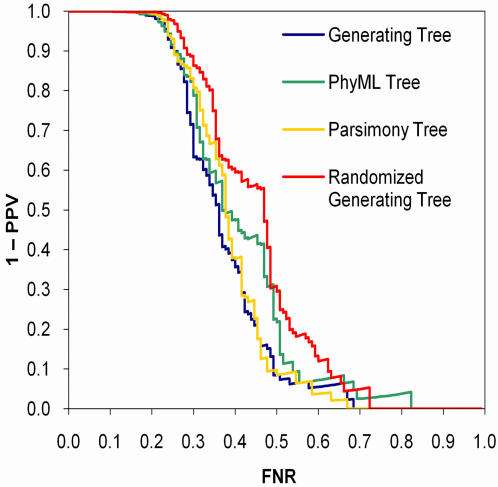
Discrimination curves for conditional models based on different trees applied to synthetic conditional influence data.

**Figure 8:**
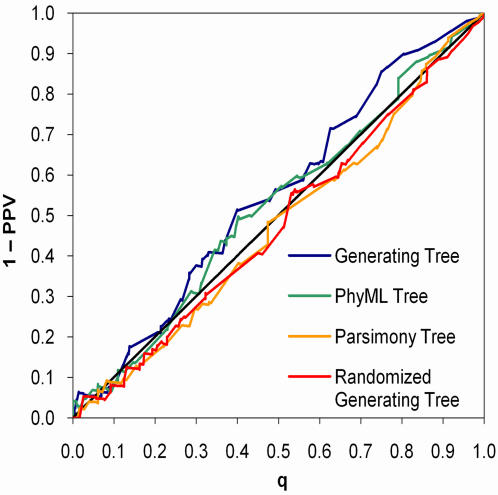
Calibration of *q*-values for conditional models based on different trees applied to synthetic conditional influence data.

As expected, the conditional model performed best using *T_gen_*, though the discrimination curves were not significantly different from those of *T_M_*_***L***_ and *T_pars_*, indicating that the conditional model is robust to variations of the tree on this data set (Figure 7). Importantly, although the discrimination curve was significantly worse using *T_rand_* rather than *T_gen_* (*p* = 0.016), the conditional model was calibrated on all four trees, indicating that, on this data set, poor trees resulted in a loss of power but not in an inflation of false discovery rates (Figure 8). This point is reinforced by the number of associations identified at *q*<0.2 for the different methods: *T_gen_* yielded eighty nine predictions whereas *T_rand_* yielded only sixty five, *T_ML_* yielded seventy eight, and *T_pars_* yielded eighty two. The positive predictive value for these predictions ranged from 0.80 to 0.85.

Although it may seem counter-intuitive that the randomized tree could find any associations, we note that the problem with the conditional model using *T_rand_* is analogous to that of an IID model. Namely, whereas an IID model will over or under count observations by not accounting for hierarchical structure that exists in the data, a randomized tree will over or under count observations by assuming false hierarchical structure. In addition, the conditional model can compensate for a tree that fits the target data poorly by setting the mutation rate to infinity and thereby assuming the data to be IID. Indeed, the median λ value under *T_rand_* was an order of magnitude higher than that under *T_gen_*.

### Application 1: Effect of immune pressure on HIV evolution

To investigate the effects of immune pressure on HIV evolution, Moore *et al.* [33] obtained HIV sequences from 234 individuals along with the HLA-A and HLA-B alleles of the infected individuals. Performing several analyses, none of which explicitly incorporated phylogenetic structure, they found strong correlations between the presence or absence of amino acids at particular positions and the presence or absence of particular HLA alleles in the infected patients, presumably reflecting the “escape” of amino acids under immune pressure. In Bhattacharya *et al.* [12], we analyzed a similar data set (N = 96, MHC-I and MHC-II alleles) and showed that use of the conditional model substantially improved the accuracy of identification of such amino-acid-HLA associations. Here, we analyzed a superset (N = 205) of the data used in [12] (MHC-I alleles only) using both the conditional and undirected joint model.

First, we constructed a phylogenetic tree from the full set of sequences (see Methods). We then used the single-variable model to determine whether any HLA alleles followed the tree. We found that two pairs of HLA-1 alleles—B*4201, Cw*1701, and A*0207, B*4601, where each pair is in tight linkage disequilibrium—followed the tree and thus separated the HLA data into two sets: (1) “C1701” consisting of these four alleles and (2) “notC1701” consisting of the remaining alleles, and analyzed these two sets separately.

Our results using BIC show that the conditional model better explains the notC1701 data, (*p* = 0.0001, *N* = 256296 ) whereas the undirected joint model better explains the C1701 data (*p* = 1.9×10^−24^, *N* = 5664). In the case of the C1701 data, it seems that the phylogenetic tree is more a confounder of the data in the traditional sense, wherein the tree is associated with both the HLA data and the sequences and induces false correlations between HLA allele and sequence.

**Figure 9:**
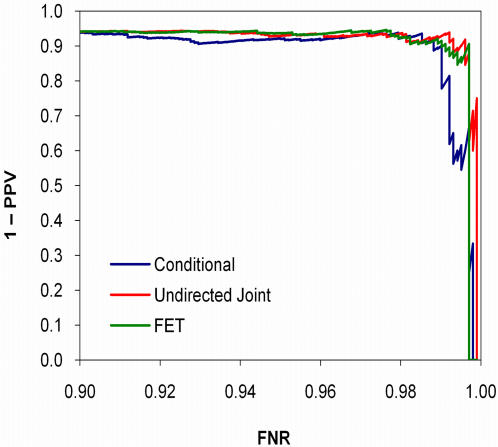
Discrimination curves for the real HLA-amino-acid data. Ground truth was estimated by identifying known epitopes within three residues of the predicted association.

In this application, we were fortunate that additional information was available to help confirm the HLA-sequence associations that we found. In particular, a known epitope in the vicinity of a found association supports the validity of that association, as immune pressure is focused on epitopes and the immediate surrounding regions that participate in the presentation of the those epitopes on the HLA molecules at the cell surface [38]. Thus, we constructed discrimination curves where an HLA-sequence association was considered “true” if it is within three amino acids of a known epitope (as described in the database at www.hiv.lanl.gov as of July 2006) with the corresponding HLA allele and “false” otherwise. This “bronze” standard does not take into account undiscovered epitopes or linkage disequilibrium, but should nonetheless be unbiased with respect to a comparison of the alternative methods for identifying associations. The discrimination curve in Figure 9 for the notC1701 data is consistent with the BIC and synthetic results, indicating that the conditional model best fits this data. We could not construct a discrimination curve for the C1701 data, as there are no known A*0207, B*4201, B*4601, or Cw*1701 epitopes in Gag.

The associations found by the conditional model with *q*<0.2 on the real data are shown in Table 1.

**Table 1:** Predicted HLA-amino acid associations in Gag.

Pos	HLA	p	q
242	B*5701	4.3E-08	<0.03
28	A*0301	1.5E-07	<0.03
242	B*5801	3.2E-06	0.03
147	C*0602	5.0E-06	0.03
26	C*0303	6.9E-06	0.05
482	B*4001	2.8E-05	0.10
397	A*3101	3.8E-05	0.13
495	B*4701	6.9E-05	0.17

### Application 2: Pairwise correlations between amino acids in HIV

Identification of pairwise correlations between amino acids is important to many areas of biology, as correlations can indicate structural or functional interaction [11], [39]. Many methods, including the undirected joint model [5], have been developed to identify correlated residues.

Continuing our focus on HIV, we applied both the undirected joint and the conditional model to the sequence data from the Western Australia cohort [33]. We concentrated on the HIV-1 p6 protein, which is cleaved from the Gag^55^ polyprotein. This fifty two amino-acid protein was chosen because it is the shortest HIV protein, making pair-wise amino acid tests feasible for all models. We fit the conditional model in both directions (making both *X* and *Y* target variables), and selected the best model according to BIC.

Remarkably, the BIC scores of the conditional model are significantly higher than those of the joint model (*p*<10^−100^, *N* = 52767). We suspect that the conditional model may be better because many mutations could be compensating for other mutations driven by HLA immune pressure. The conditional model finds that 893 of the 52767 (1.7%) amino acid pairs, and 310 of 1300 (24%) of position pairs, are correlated at *q*<0.2. This dense network of interactions is consistent with the idea that many of the mutations are compensatory in nature. For example, the conditional model identifies two HLA-mediated escape mutations in p6 (Table 1). Mutations at these two positions account for forty two (13.5%) of the position-pair correlations.

**Figure 10:**
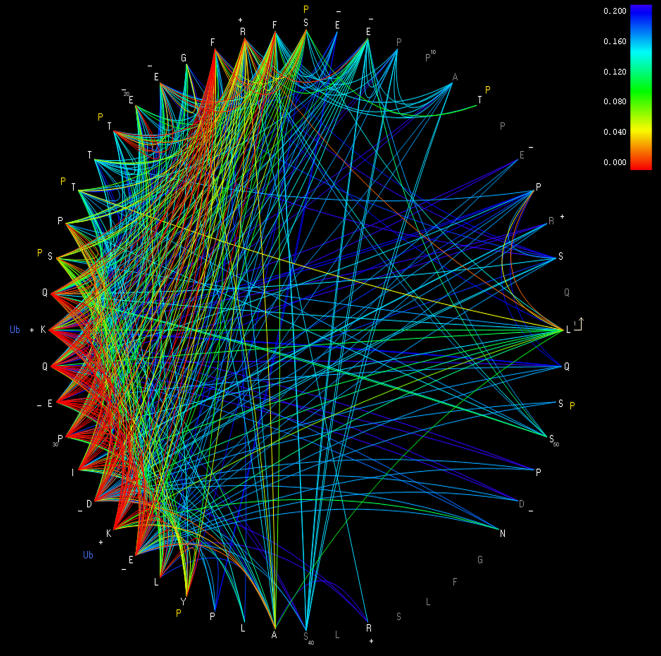
Correlated amino-acid pairs in HIV-1 p6. The fifty two consensus amino acids of P6 are drawn as a circle, with the N-terminal end shown at the far right and the protein extending counter-clockwise. Each arc represents an association predicted by the conditional model that is significant at *q*<0.2. Arc color reflects the *q*-value of the association. Dark gray residues denote positions where there were fewer than three sequences with a non-consensus residue. The associations used to construct the figure are available as Dataset S1. Annotations of individual residues are: P, phosphorylated residue; Ub, site of ubiquitinization; +/−, charged residue.

We have developed a tool for visualizing the network of dependencies (Figure 10). The visualization highlights at least one potentially interesting set of interactions. In particular, R16 is strongly correlated with residues at positions 21 through 36, many of which are correlated with each other as well as with residues throughout the protein. This complex network of interactions connects the two α-helix domains of p6 [40] and may be of structural or functional significance.

### Application 3: Genomic search for genotype-phenotype associations in Arabidopsis thaliana

Aranzana *et al.* recently demonstrated the potential utility of genome wide association (GWA) studies, as well as the importance of accounting for hierarchical population structure [23]. In this study, the authors genotyped 848 loci in ninety six *Arabidopsis thaliana* strains and looked for haplotypes that were correlated with hypersensitive response to *P. syringae* strains expressing one of three avirulence (avr) genes (avrRpm1, avrRpt2, or avrPph3). In plants, each avr bacterial protein is recognized by a corresponding resistance (R) gene. If both plant and pathogen have active copies of the respective avr-R genes, a biochemical cascade is triggered at the point of infection, leading to massive programmed cell death and containment of the infection (for review, see [41]). Using both an IID-based model and a method that used the hierarchical population structure that was constructed from the sequenced loci, the authors showed that loci adjacent to the known R genes are highly correlated with the corresponding avr phenotypes. Unfortunately, the authors noted that their statistics were poorly calibrated, precluding confident predictions of the other pathogen-response proteins that are involved in the hypersensitive response cascade. Here, we apply our well-calibrated methods to the same data, using a genetic-similarity tree constructed from the sequence data.

Although *Arabidopsis* is a sexually reproducing species, it is highly selfing, meaning that organisms primarily mate with themselves. As a result, the population structure induced is hierarchical and bears striking resemblance to a phylogenetic tree. Aranzana *et al.* found that a tree built from pairwise similarity matrices on shared alleles provided a good qualitative description of both the geographic distribution of the organisms and the distribution of avr and flowering time alleles [23]. Quantitatively, we found that sixty one percent of the haplotypes and two of the three phenotypes followed the “phylogenetic” tree constructed from the sequence data.

When applying our conditional model to this application, it is not clear whether the target variables should be haplotypes or phenotypes. In general, genetic variations directly influence phenotypes, but phenotypes also indirectly influence haplotypes through selection pressure. As two thirds of both variables followed the tree, we ran the conditional model in both directions, once using the phenotypes as the target and once using them as the predictor, using BIC to determine which direction was best for any given haplotype-phenotype pair.

**Figure 11:**
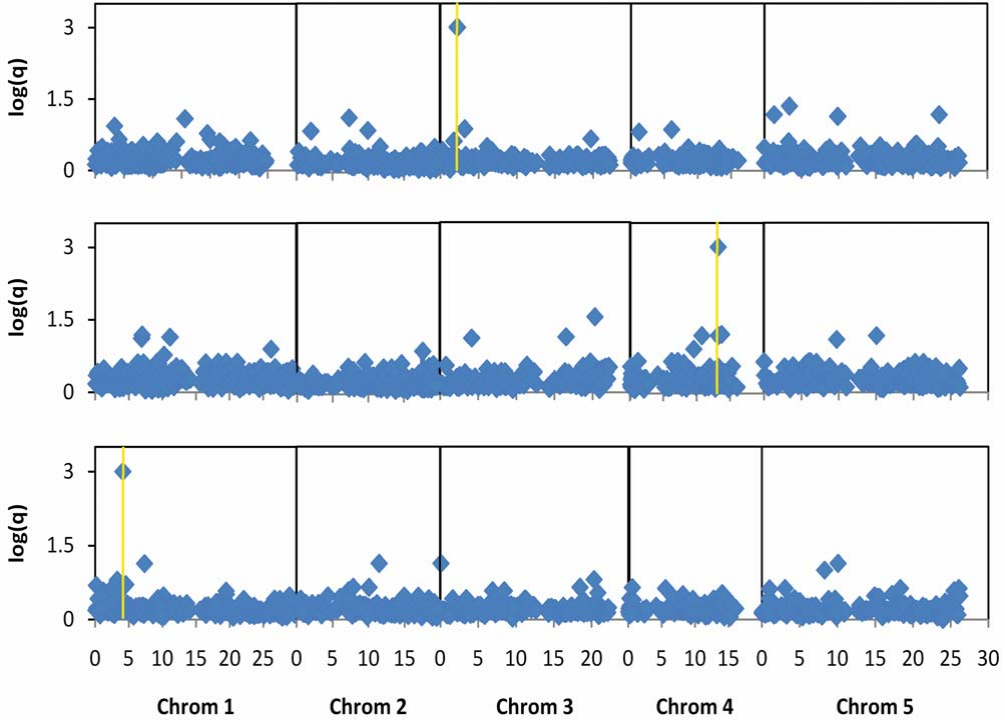
Genomic distribution of genotype-phenotype association scores for *Arabidopsis* bacterial response. 4681 haplotypes were compared against each of the three bacterial response phenotypes, Rpm1 (top), Rpt2 (middle) and Pph3 (bottom). For each haplotype, the four conditional models were run and negative log_10_ of the most significant *q*-value is plotted. For each phenotype, the most significant association is a locus within 10 kb of the corresponding R gene (yellow lines). The dotted line shows the *q* = 0.2 threshold.

We found that the BIC scores for the conditional and undirected joint models were not significantly different (*p* = 0.70, *N* = 14043). Consequently, we arbitrarily choose to examine the results of the conditional model in detail. Figure 11 shows the genome wide distribution of conditional influence *q*-values for each of the three phenotypes. For each phenotype, the most significant association is a locus near the corresponding R gene. We constructed this figure to be similar to the one in Aranzana *et al.* to facilitate comparison.

Our synthetic tests indicate that the conditional method is well calibrated, implying that roughly 80% of the associations we find with a *q*<0.2 cutoff should be legitimate. To explore this implication, we took the fifty one genotypic associations (comprising forty unique loci) that correlate with these three hypersensitive phenotypes at this cutoff, and noted which of the associated loci were near known or putative bacterial response proteins according to http://www.arabidopsis.org. Our standard of “true positive” was defined to be proximity to a protein whose description included the phrase “disease response” (see Methods). We found that twenty three (45%) of the predictions were within fifty kilobases (kb) of such proteins. This “bronze” standard undoubtedly contains false positives and false negatives, and therefore cannot be used to confirm that our methods our calibrated. Nonetheless, we easily can reject the null hypothesis that these twenty three associations are found near disease-response proteins by chance (*p*<0.0001).

### Related work

Evolutionary biologists have long been interested in studying correlated traits in the midst of population structure due to phylogenetic relationships (for reviews, see [42], [43]). For association studies on continuous variables, the method of choice has been to use independent contrasts [1]. In this method, the data are assumed to be derived from a Brownian motion (or other Gaussian) model of evolution and the *n* samples are converted to *n*−1 differences between adjacent nodes in the tree, with the variance of the differences computed according to the branch lengths of the tree. The resulting differences are independent, allowing for regression tests between two traits. This method is effective and well used, but is appropriate only for continuous data. For discrete data, early work focused on reconstructing ancestral states using parsimony and looking for correlations between transitions [2], [3]. Pagel [4] presented a maximum likelihood approach that averages over all possible configurations of the internal nodes. Pollock, Taylor and Goldman [5], which we study here, is a reversible version of Pagel's method.

Population structure in biological data has also been addressed in the area of GWA studies. There are rather different approaches in this community that have been used to compensate for population structure. A more commonly used approach attempts to recalibrate standard statistics by normalizing results according to the distribution of the statistic across the entire genome [24], [25]. As we have seen, solutions to calibration are insufficient, as population structure also affects discriminatory power. Another approach assumes population structure is flat and can be captured by a small number of (perhaps overlapping) clusters or continuous hidden variables [26]–[30]. Although these methods increase discriminatory power relative to simple IID models, there is mounting evidence that population is hierarchically structured. For example, in addition to high level geographical/social constraints, structure exists within a number of subpopulations that have been studied [19]–[22]. It stands to reason that, if hierarchical models describe the data better than flat cluster models, then such models will have higher discriminatory power. Thus, several authors have suggested that a more accurate approach would be to model population structure hierarchically [23], [31], [32]. Aranzana *et al.* described one such model in their *Arabidopsis* study. As mentioned, however, they found that their model was not calibrated and their data precluded a discriminatory power analysis. Yu *et al.* extended the linear mixed-effects model of Kennedy *et al.* [31], in which hierarchical population structure is modeled with random effects. The approach is well calibrated, but is typically inappropriate for discrete data.

Similar to what happened in the GWA community, those studying amino acid coevolution initially ignored the phylogenetic structure of the protein sequences [44]–[47], but came to recognize that phylogeny played a confounding roll [10], [48], [49]. A number of methods emerged that attempted to calibrate *p*-values based on global or local measures of average similarity [6]–[9] or flat clusters based on early bifurcations of the phylogenetic tree [11]. One approach that explicitly incorporates the phylogeny is the parametric bootstrap, in which standard IID models are recalibrated by generating independently evolved amino acids according to the single variable model [10], [11]. On our synthetic coevolution data, we found this approach to be too severe, resulting in overly conservative *q*-value estimates (see Figure 5).

## Discussion

We have identified two evolutionary processes that can confound association analyses and have defined two corresponding generative models for discrete data that can correct for and even leverage the existence of these processes. We have found that explicitly modeling evolutionary processes increases discriminatory power and results in well-calibrated estimates of one minus positive predictive value. We have implemented methods for fitting these models to data and a tool for visualizing the results of the analysis. These tools are available on the internet.

The undirected joint model assumes that the two variables coevolve such that a mutation event in either variable can elicit a corresponding change in the other variable. In contrast, the conditional model assumes that a single variable is distributed according to the tree and is influenced by the predictor variable only at the tips of the tree. Of course, other evolutionary processes are possible. In Methods, we describe a third process in which variables coevolve, but only one variable influences the evolution of the other.

Neither the undirected joint nor conditional model outperformed the other on all real data sets, suggesting that both models should be considered when analyzing new data. Nonetheless, the conditional model better fit most of the real data that we analyzed. The conditional model better described the effects of immune pressure on HIV evolution, and perhaps more surprising, better described the correlation between HIV-1 p6 amino-acid pairs. This observation may be due to the rapid evolution of HIV and positive selection pressure from the immune response in conjunction with compensatory mutations in the observed sequences.

Our study has focused on the correlation of discrete (specifically binary) variables. Generalization to multistate variables is straightforward, although may suffer a loss in power. Our methods can also be generalized to continuous and/or discrete predictor and target variables. When the target variable is continuous, the conditional model is a special case of a linear mixed-effects model (K.M. Kang, N. Zaitlen, C.M. Wade, A. Kirby, D.H., M.J. Daly and E. Eskin, submitted). The conditional model can also be generalized to situations with multiple predictor and target variables, thus producing a directed network (acyclic or otherwise) of relationships among multiple variables. Potential applications of multiple predictor variables include learning the combined effects of drug and immune pressure on HIV evolution, identifying chains of compensatory mutations, learning the influence of diploid genes on phenotype, and learning networks of interacting genes and proteins. Finally, one could also use the undirected joint or conditional models to learn the structure of phylogenentic or hierarchical relationships rather than learning the tree structure with standard methods that ignore correlations.

The problem of population structure confounding association studies is a ubiquitous problem across many biological disciplines. Existing solutions vary across these disciplines, but typically focus on correcting for shared population structure. As we have seen, however, population structure in either variable can lead to loss of discriminatory power and poor statistical calibration. The flexibility and intuitive nature of generative models makes them a natural and powerful choice for dealing with a variety of biological processes.

## Methods

### Data sets

We obtained HIV aligned sequences and HLA data from the Western Australia cohort (HIV sequence accession numbers AY856956–AY857186 and EF116290–EF116445) [33], [50]. We noted some anomalies in the alignment of p6 and corrected them by hand. We constructed the phylogenetic tree for Applications 1 and 2 from the full Gag DNA alignment by applying PHYML [51] with the general reversible model, optimized tree topology, maximum likelihood estimates for the base frequencies, estimated proportion of invariable sites, and four substitution rate categories with an estimated gamma distribution parameter. When identifying associations, we binarized amino acids, such that a single association test compared the presence or absence of the residue in question. Ambiguous codons were treated as uncertainties. For example, if a codon was *X* or *Y*, then it was treated as unknown for codon *X*, but known to be false for any codon not equal to *X* or *Y*.

The Arabidopsis data set was taken from Aranzana *et al.* [23]. We built the genetic-similarity tree using PHYML from a set of sequences consisting of positions in the locus alignments for which at least two sequences differed from the consensus. Following [23], for each genotyped locus, we defined haplotypes according to sequence identity after removing positions for which only one sequence varied from the consensus. All sequences that accounted for less than five percent of the data were clustered together.

#### Synthetic data sets

We generated synthetic data to approximate real data as closely as possible. In the case of synthetic conditional influence, we first ran the conditional model on the real HLA data set to obtain reasonable parameter values. To generate predictor variables, we permuted the real HLA alleles to ensure the data were IID. For each association in the real data set, we generated synthetic target data in one of two ways: (1) if the association was significant (*q*<0.3), we took the corresponding HLA allele and the parameters learned on the real allele-amino acid pair and generated data for the target variable; otherwise (2) we generated the target variable data using the parameters learned by the single-variable model. Because there were only twenty six significant associations, we generated five synthetic associations for each real association. For each replicate, we chose a different HLA allele, using the five HLA alleles whose frequencies most closely matched that of the real HLA allele on which the association was based.

In the case of coevolution, we first fit the undirected joint model to the HIV p6 data set, and then generated synthetic associations using the learned parameters. For these data, we generated one synthetic association for each real association (*q*<0.3). For all non-significant real associations, we generated the predictor and target variables using the single-variable model with the parameters learned by that model on the real data.

#### Inferring trees from synthetic data

When analyzing the sensitivity of results to tree structure, we needed to infer a phylogenetic tree from synthetic data. To do so, we constructed binary sequences from the synthetic target variables, such that each position in the binary sequence corresponded to a target variable. We then used the PHYML software as described above for real data to infer a maximum likelihood phylogeny. In addition, we used the dnapars program from the PHYLIP package [52] to infer a phylogeny using parsimony. We ran dnapars using default settings, with the exception that only a single tree was optimized.

### Computing q-values

The independence (null) models are nested inside the undirected joint and conditional models and contain one less parameter. Therefore, the asymptotic distribution of the log-likelihood ratio is χ^2^-distributed with one degree of freedom from which *p*-values can be computed. Simple *p*-values are, however, of limited use in the context of high levels of multiple hypothesis testing. Whereas a Bonferroni correction could be used to limit false positives, the high number of tests (typically in the tens or hundreds of thousands) forces an almost complete loss of power. Consequently, we used *q*-values to control for multiple tests. To compute a *q*-value, we first computed a False Discovery Rate (FDR), which measures the expected fraction of true positives for any given *p*-value threshold *p*_0_ [53]. For a given *p*_0_, we estimated FDR to be the ratio of the expected number of associations with *p*<*p*_0_ under the null distribution to the observed number of associations with *p*<*p*_0_ in the real data. In our experiments, we generated ten associations under the null model for each examined *X–Y* pair to compute the expectation under the null. (As described by, e.g., Storey and Tibshirani [54], the true FDR should be computed using null data that is generated only from observed values from which clearly non-null data have been removed. As only a small number of tested associations were real in our applications, the inclusion of null data generated from these associations introduced a small, conservative bias in our computation of FDR.) We then computed the *q*-value as the minimum FDR seen for *p_i_*≥*p*_0_ [54]. This guaranteed *q* to be a monotonic increasing function of *p*_0_. (In general, FDR is expected to be a monotonic function of *p*_0_, but is rarely monotonic in practice due to, e.g., variance in the statistic.)

#### Generating null data

When testing for an association between variables *X* and *Y*, null data can be generated in one of four ways: permute the observations of *X*, permute the observations of *Y*, parametrically bootstrap observations for *X*, or parametrically bootstrap observations for *Y*. The best method—the one that most accurately constructs the null data—will depend on the data set being analyzed. For example, suppose we have data where both the predictor and target variable follow a given tree. Here, we should parametrically bootstrap observations for either the target or predictor variable, as doing otherwise would produce data that no longer follows the tree, introducing a bias in the null data. In contrast, suppose observations of the predictor variable are IID but observations of the target variable are not well described by the tree. Here, we should permute the observations of the predictor variable because (1) they remain IID under permutation and (2) a parametric bootstrap of the target variable under the assumption that the data follows the tree would produce biased data. The best method for generating null data may also depend on the model being used to analyze that data as the undirected joint and conditional models can be sensitive to different biases in the data.

We have developed and used a systematic approach for determining which null-generation method to use for a given data set and given model for analysis based on two observations. First, as the computation of *q*-values depends on the distribution of *p*-values under the null hypothesis, it was important to select a null-generation method that produced an accurate distribution of *p*-values. Second, in all the data sets that we analyzed, the vast majority of variable pairs were not associated—that is, they satisfied the null distribution. Consequently, given a data set and a given model for analysis, we chose the data-generation method by identifying the one that yielded a distribution of *p*-values that most closely matched that produced by the given (real) data.

Our approach yielded the following choices: permutation bootstrap of the predictor variable for Applications 1 and 3, and parametric bootstrap of the predictor variable for Application 2. For the analysis of synthetic data, we used null-generation methods that would preserve the known distributions of the predictor and target variables: permutation bootstrap of the predictor variable for the conditional influence data set, and parametric bootstrap of the predictor variable for the coevolution data set.

#### Computing *q*-values on subsets of data

When computing *q*-values, it is sometimes useful to partition the tests into two or more sets to obtain more informative values. For example, suppose we partition a set of tests into two sets *A* and *B* corresponding to positive and negative correlations, respectively. For simplicity, assume the null distributions underlying *A* and *B* are identical. If *A* and *B* have the same number of associations with *p*<*p*_0_, then we expect *q_A_*(*p*_0_) = *q_B_*(*p_0_*) = *q_A_*_∪*B*_(*p*_0_). If *A* has a higher number of associations with *p*<*p*_0_ than does *B*, however, then we expect *q_A_*(*p*_0_)<*q_A_*_∪*B*_(*p*_0_)<*q_B_*(*p*_0_). That is, computing *q*-values with respect to the merged set of associations will inflate the *q*-values of the associations in *A* and deflate the *q*-values of the associations in *B*. Whereas *q* is still the expected number of false positives, computing *q_A_*_∪*B*_(*p*_0_) assures that the majority of those false positives are in *B* and some true positives in *A* are likely to be lost. The disadvantage of partitioning the tests is that more null data must be generated in order to preserve the variance of the estimate for *q*.

In this work, we split our tests along natural boundaries. In our experiments with the conditional model, we computed *q*-values separately for each possible transition matrix (escape, attraction, reversion, and repulsion). For the undirected joint model and when computing FET, we computed *q*-values separately for positive and negative correlations.

### Performance Evaluation

#### Comparing discrimination curves

To compute the significance of the difference between the discrimination curves of two methods *A* and *B* applied to synthetic data, we computed the areas under each curve and tested whether the area under *A*'s curve was significantly different from the area under *B*'s curve.

The permutation test was carried out as follows. Given a set *T* of *n* association tests, a method *A* induces an ordering *T^(A)^* over *T* such that *t_i_* ^(*A*)^<*t_j_* ^(*A*)^(*i*≠*j*) implies that *t_i_* is more significant than *t_j_* by method *A*. Let **a** = [*a*_1_, *a*_2_,…,*a_n_*] be a binary vector such that *a_i_* = 1 if and only if *t_i_* ^(*A*)^ is an association. We computed the area under the curve (AUC) of *A* as a function of **a**.

When comparing the discrimination curves of two methods *A* and *B*, the null hypothesis is that the curves come from equivalent methods, such that the *i*-th prediction of *A* is just as likely to be an association as is the *i*-th prediction of *B* (that is, *Pr*[*a_i_* = 1] = *Pr*[*b_i_* = 1]). We estimated this null distribution using a permutation bootstrap, in which we randomly swapped the assignments of *a_i_* and *b_i_* and recomputed *AUC*(**a**)–*AUC*(**b**). (For a two-tailed test, we used the absolute value of the difference.) With synthetic data, we have prior knowledge as to which method will perform best (the model used to generate the data). Thus, we report the one-tailed *p*-value.

#### Evaluating performance on *Arabidopsis thaliana* GWA study

To determine whether a set of predictions was likely to be enriched for disease response proteins, we downloaded the genomic positions of all genes whose description contained the phrase “disease response” (data taken from http://www.arabidopsis.org). There were 226 such *target genes* matching this search criterion, including the known R genes, genes that are known to be involved in the hypersensitive response cascade and a number of putative proteins with high sequence similarity to the known R genes. For each predicted locus, we calculated the minimum distance to one of these genes. We used the fifty kb threshold because it is the most conservative estimate of linkage disequilibrium [55] and because, at higher distances, it becomes unsurprising that a locus is proximal to one of the 226 target genes.

We computed the probability that *m* of the *n* predictions would fall within fifty kb of some target genes using a permutation bootstrap. In each iteration of the bootstrap, we randomly selected (without replacement) *n* haplotypes from our study and counted how many were within fifty kb of a target gene.

### Directed joint model

The Pollock model for coevolution assumes a symmetric or undirected relationship between two coevolving variables *X* or *Y.* Alternatively, we can imagine a *directed joint model*, wherein *X* or *Y* coevolve, but *X* influences the evolution of *Y* but not vice versa. In this process, *X* evolves as in the single-variable model, and *Y* evolves with a rate that depends on whether *X* is absent or present. In particular, the transition probability matrix for the model is given by
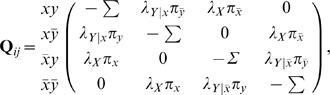
(3)where the diagonal entries assure that the rows sum to 0. This model, like the undirected joint model, has five parameters: π*_x_*, π*_y_*, λ*_X_*, λ*_Y_*_|*X*_, and λ*_Y_*_|*X̅*_. As in the undirected case, we compute the probability that one instance of *XY* transitions to another along a branch of length *t* by determining **P** = exp[**Q** *t*], using standard numerical Eigen-decomposition techniques [36].

## Supporting Information

Dataset S1 HIV-1 p6 amino acid pairs that are correlated at q<0.2.
(0.36 MB XLS)
